# Interstate Medical Representation

**Published:** 1888-04

**Authors:** 


### INTER-STATE MEDICAL REPRESENTATION.

It is gratifying to observe the growing tendency, year by year, towards a general interchange of delegates between State Medical Associations; and it should be encouraged, and made a permanent feature. For some years the Texas State Medical Association has been in the habit of appointing a corresponding delegate, and last year, delegates were appointed to the Missouri State Medical Association. Such a course could but redound to the good of all. It would promote a more intimate acquaintance amongst medical men, and engender kindly feelings; and above all, it would be mutually beneficial. Our system of organization, and our plan of conducting the meetings are by no means perfect; nor can it be said that any State Association has attained perfection in those regards; but some are better than others, while all, perhaps, possess some good features. By a general interchange of delegates, each Society could, in time, learn the points of advantage in the organization and working of others, and thus each could better its own.

Last year the Alabama State Medical Association honored us by sending one of her most distinguished Fellows to represent Alabama on the floors of the Texas convention, and it was a source of much gratification. It would have seemed but natural and right, that this particular courtesy should have been reciprocated, but doubtless, in consequence of many distracting influences, it was overlooked by the nominating committee. Later, it was understood that the President of the Alabama Association particularly wished it, and President Burroughs, taking the correct view of the subject, and assuming that it was an oversight on the part of the nominating committee, although, strictly speaking, he had no authority to do so, made an appointment. He appointed Dr. J. D. Osborn. Dr. Osborn is a native of Alabama, and is well and favorably known to the profession of that State, and the appointment was an eminently judicious one, and it is to be regretted that the Doctor could not accept it. The President then tendered the appointment to Dr. Daniel, the Secretary; but in the very nature of things, the meetings occurring so near together, it was impossible for him to serve, and thus Texas will have no representative at the approaching meeting of the Alabama State Association. It should be stated that the question was sprung only a short while ago, and it is not to be expected that any one could have been found to accept the appointment on only a few days' notice.

It is generally conceded, we believe, that the Alabama Association is one of the best medical organizations in America. It is a chartered institution, and has the power of making appointments on the State Board of Health and to other medical offices, collects and registers the vital statistics, runs the quarantine, etc. Indeed, the Alabama State Board of Health is the Medical Department of the State government, and the Constitution of the State Association is part of the organic law of the State,—a status of the medical profession, with reference to the government, which it has been the aim and ambition of the JOURNAL to secure for the Texas profession. We have always held that the medical profession, by virtue of its learning and numbers, should be recognized; that they are the best, and only judges of what sanitary laws are needed, and if not empowered to make them, they should at least be consulted on the subject, by those who do make them; therefore, a State Board of Health in Texas is a necessity in our judgment, and it should be an Advisory Board to the Governor and Legislature; the Medical Department of the Government, in fact. There are departments of every other kind—even agriculture—why not a Bureau of Health?

Recognizing, then, the organization of the Alabama Association as a model, in many respects, and the Texas State Association being on the very eve of making radical changes in her constitution, it was eminently opportune and advisable that we should have had a delegate in attendance at the approaching meeting. An intelligent man, of popular manners could have reflected credit on the Association, and could have returned to our own meeting, possessed of many items of information which, doubtless, would have been of service in the general overhauling of our constitution; but, it is too late now; let the nominating committee "make a note on it," and fail not, in future, to appoint delegates to our sister State Societies, especially those who have honored us—paid the first visit. Courtesy requires that it should be returned.

